# Supplementary material for: Two Dimensional β-InSe with Layer-Dependent Properties: Band Alignment, Work Function and Optical Properties
Source: Nanomaterials (Basel). 2019 Jan 9;9(1):82. doi: 10.3390/nano9010082 (PMC6358860; doi:10.3390/nano9010082)
Supplement: Supplementary file 1 [file nanomaterials-09-00082-s001.pdf]

nanomaterials

Supplementary material for

# Two Dimensional β-InSe with Layer-Dependent Properties: Band Alignment, Work Function and Optical Properties

**David K. Sang [1,2], Huide Wang [1], Meng Qiu [1], Rui Cao [1], Zhinan Guo [1,*], Jinlai Zhao [1,2], Yu Li [2,*], Quanlan Xiao [1], Dianyuan Fan [1] and Han Zhang [1,*]**

[1] Shenzhen Key Laboratory of Two Dimensional Materials and Devices, Shenzhen Engineering Laboratory of Phosphorene and Optoelectronics, International Collaborative Laboratory of 2D Materials for Optoelectronics Science and Technology, College of Optoelectronic Engineering, Shenzhen University, Shenzhen 518060, China; dks@szu.edu.cn (D.K.S.); wanghuide@szu.edu.cn (H.W.); qiumeng@szu.edu.cn (M.Q.); caorui@szu.edu.cn (R.C.); zhaojl@szu.edu.cn (J.Z.); xiaoql@szu.edu.cn (Q.X.); fandy@cae.cn (D.F.)

[2] College of Materials Science and Engineering, Shenzhen University, Shenzhen Key Laboratory of Special Functional Materials, Shenzhen 518060, China

* Correspondence: guozhinan@szu.edu.cn (Z.G.); liyu@szu.edu.cn (Y.L.); hzhang@szu.edu.cn (H.Z.)

Received: 23 November 2018; Accepted: 27 December 2018; Published: date

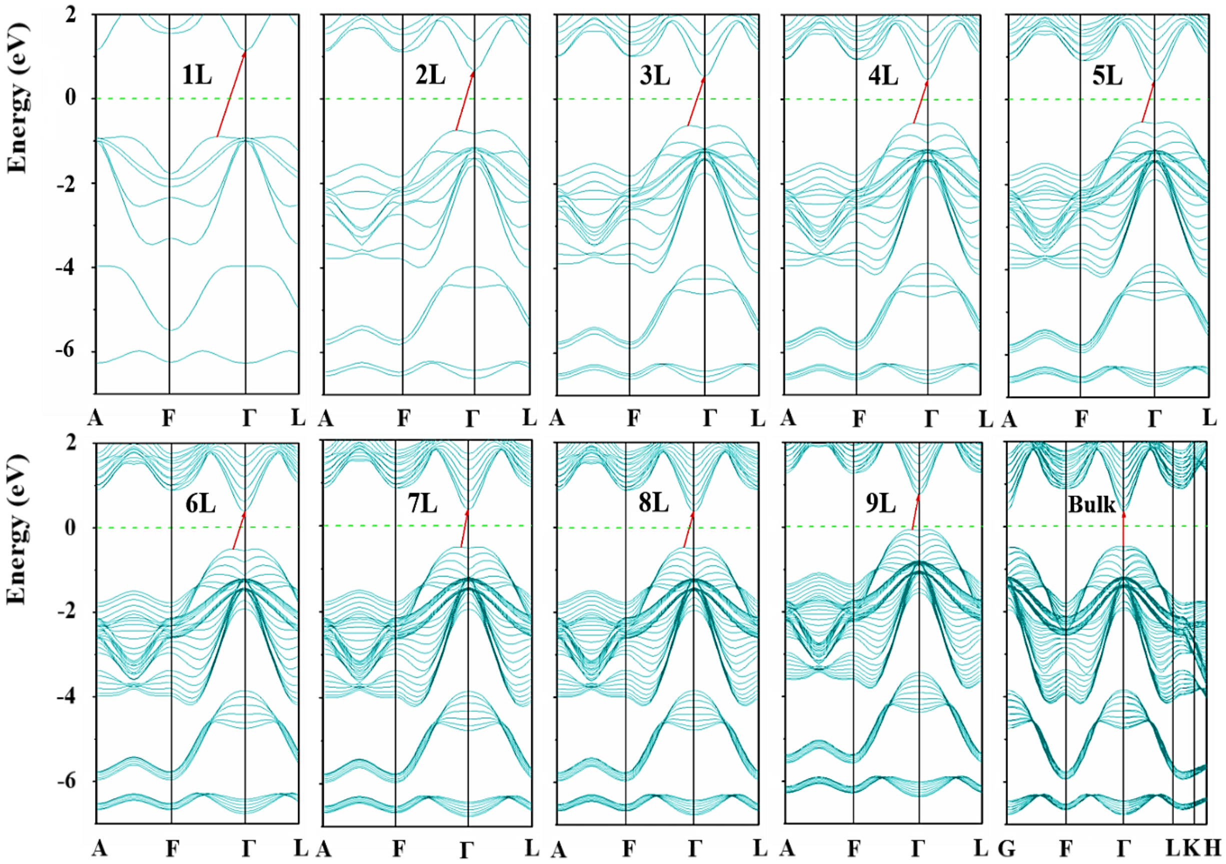


**Figure S1:** Electronic band structures of β-InSe monolayer (1L), few-layer (2L to 9L) and bulk β-InSe extracted from GGA-PBE functional calculations. The green dashed line is Fermi energy level set to 0.0 eV.

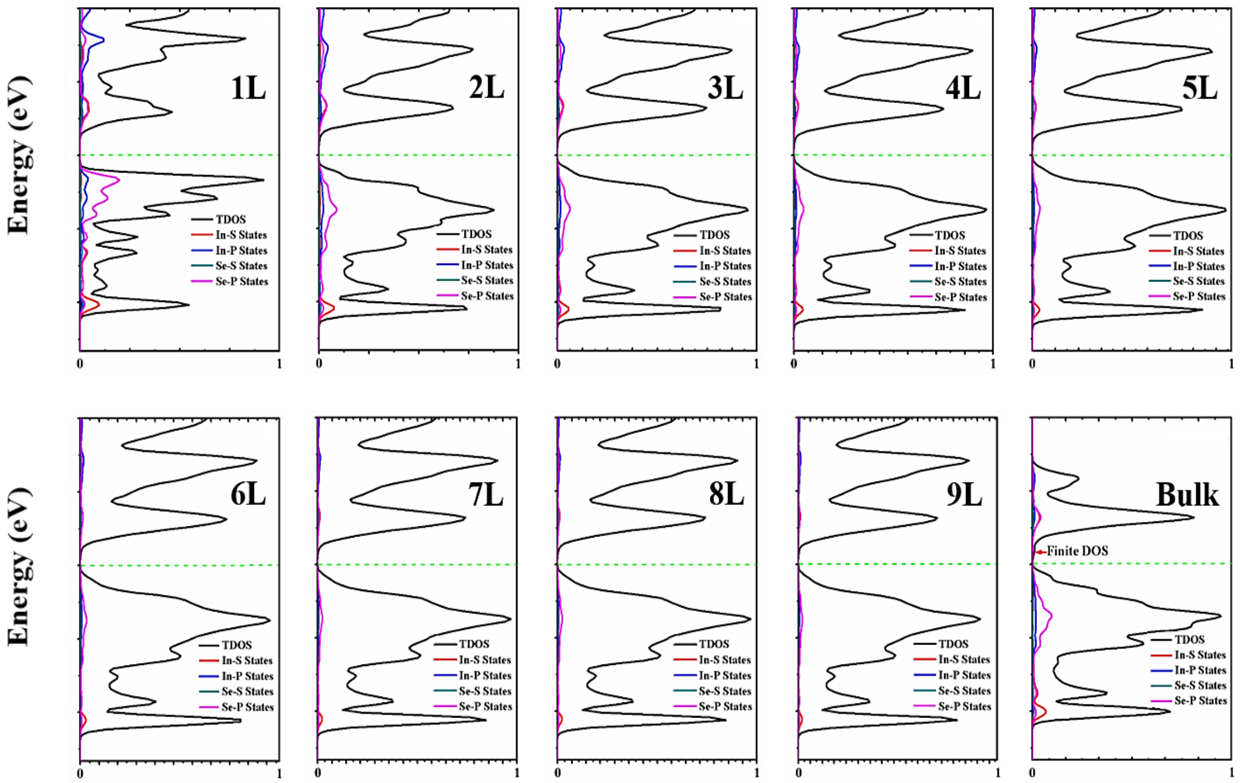


**Figure S2:** Calculated total density of states (TDOS) and partial density of states (PDOS) of β-InSe monolayer (1L), and few layer (2L to 9L) and bulk β-InSe based on GGA-PBE functional. The green dashed line is Fermi energy level set to 0.0 eV.

**Table S1:** Tabulation of band gap energy values extracted from different functional calculations of β-InSe monolayer (1L), few-layer (3L and 5L) and bulk β-InSe.

| Number of layer (L) | GG-PBE (eV) | optB86b-vdW (eV) | optPBE-vdW (eV) | HSE06 (eV) |
|---|---|---|---|---|
| 1 | 2.02 | 1.97 | 1.98 | 2.84 |
| 3 | 1.16 | 1.05 | 1.84 | 1.98 |
| 5 | 0.950 | 0.894 | 0.987 | 1.84 |
| Bulk β-InSe | 0.674 | 0.685 | 0.705 | 1.39 |

**Table S2:** Tabulation of band gap energy values extracted from DFT calculations of β-InSe monolayer (1L), few-layer (2L to 9L) and bulk β-InSe based on GGA-PBE.

| Number of layer (L) | Band gap values (eV) | Number of layer (L) | Band gap values (eV) |
|---|---|---|---|
| 1 | 2.02 | 6 | 0.897 |
| 2 | 1.40 | 7 | 0.861 |
| 3 | 1.16 | 8 | 0.840 |
| 4 | 1.03 | 9 | 0.817 |
| 5 | 0.950 | Bulk β-InSe | 0.674 |

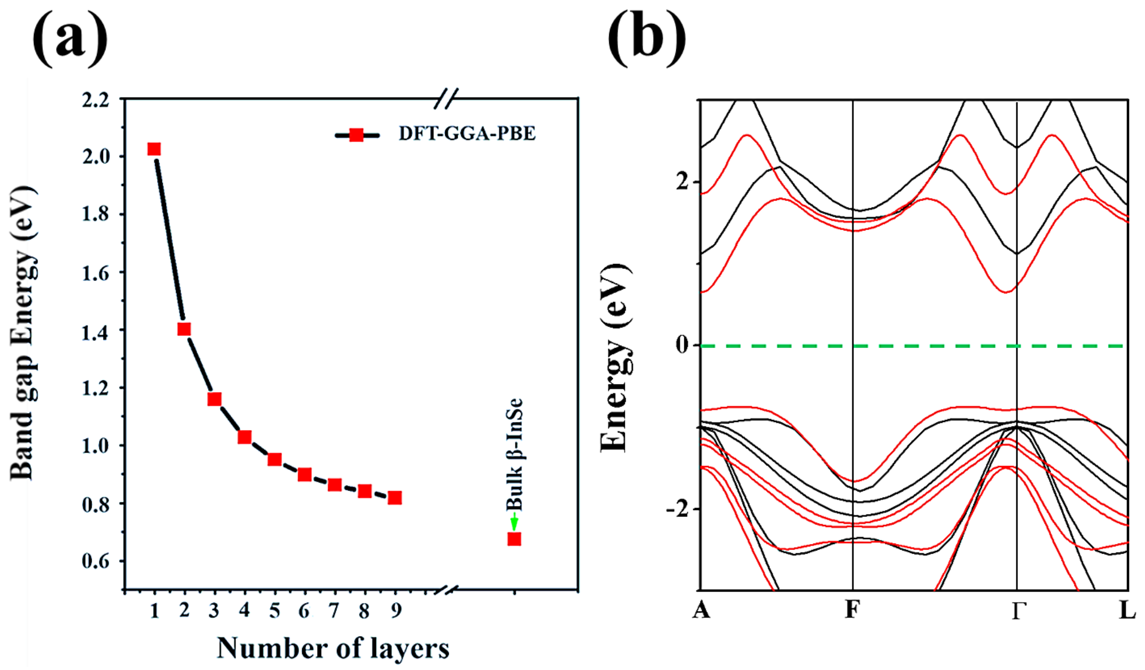


**Figure S3:** (a) GGA-PBE band gap energies of few-layer of β-InSe as a function of number of layer, (b) electronic band structure of monolayer β-InSe extracted from GGA-PBE calculations, band dispersion line with red is for band with SOC ($E_g$ = 1.40 eV) and with black is for band without SOC ($E_g$ = 1.42 eV). The green dashed line is Fermi energy level set to 0.0 eV.

**Table S3:** Tabulation of work function values of β-InSe monolayer (1L), few-layer (2L to 9L) and bulk β-InSe.

| Number of layer (L) | Work function (eV) | Number of layer (L) | Work function (eV) |
|---|---|---|---|
| 1 | 5.22 | 6 | 5.00 |
| 2 | 5.05 | 7 | 4.99 |
| 3 | 5.02 | 8 | 4.99 |
| 4 | 5.01 | 9 | 4.98 |
| 5 | 5.00 | Bulk β-InSe | 4.77 |

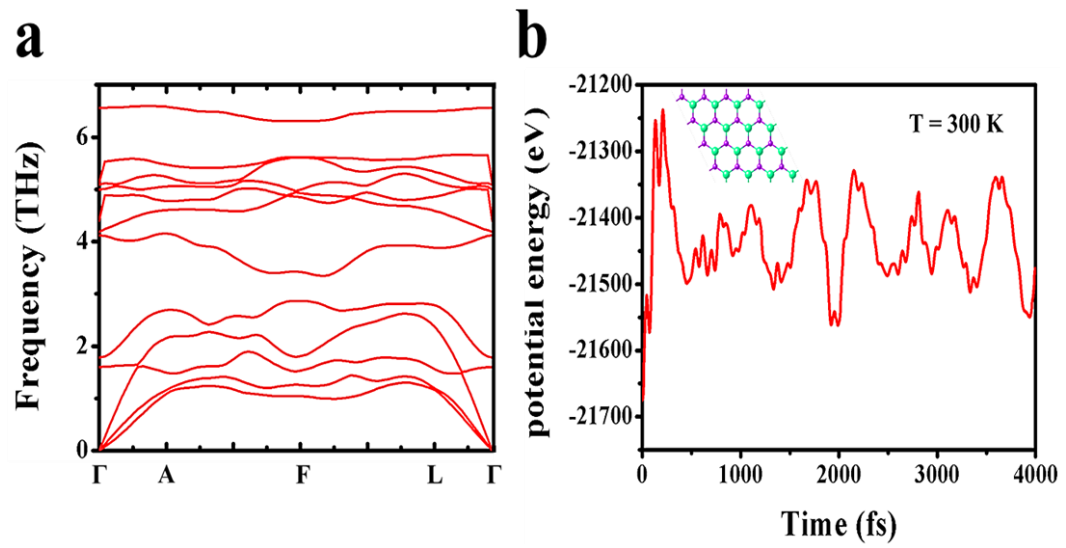


**Figure S4:** (**a**) Calculated phonon band dispersion structure of β-InSe a long high-symmetry direction Γ-Z-M-A-Γ (**b**) Total potential energy fluctuation of β-InSe monolayer from 500 to 4000 fs during AIMD simulations at the temperature of 300K.The inset show the snapshot at 1 ps

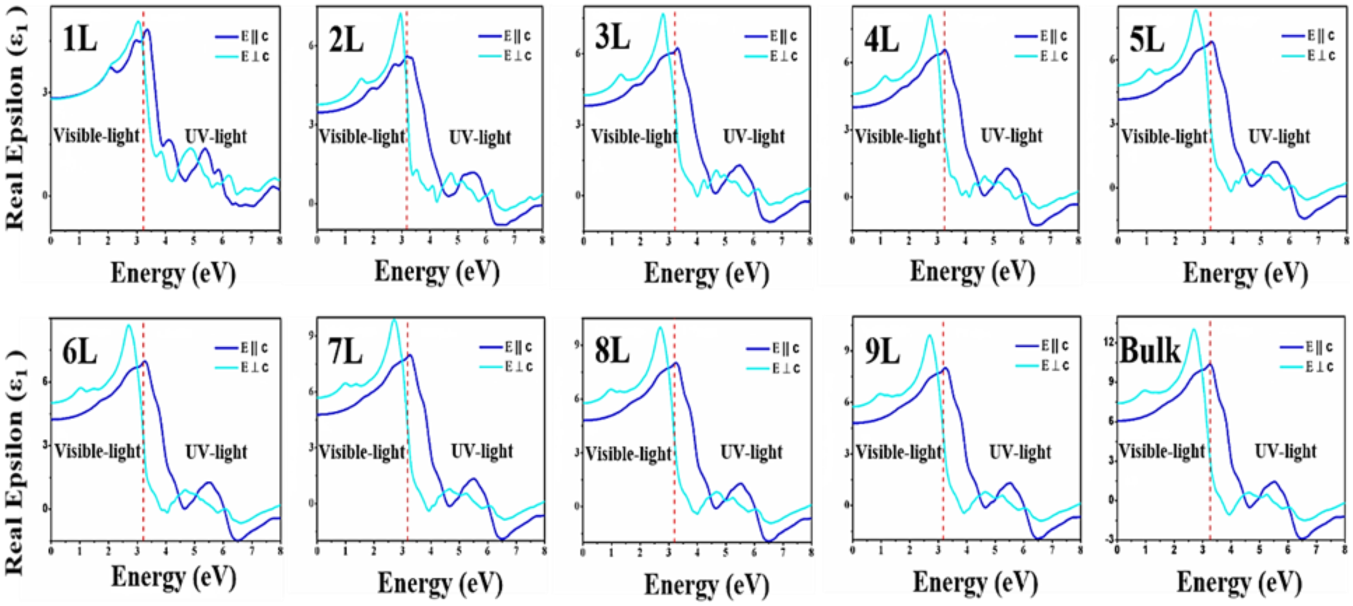


**Figure S5:** Calculated real part of the dielectric function along x and z direction for β-InSe monolayer (1L), few-layer (2L to 9L) and bulk β-InSe.

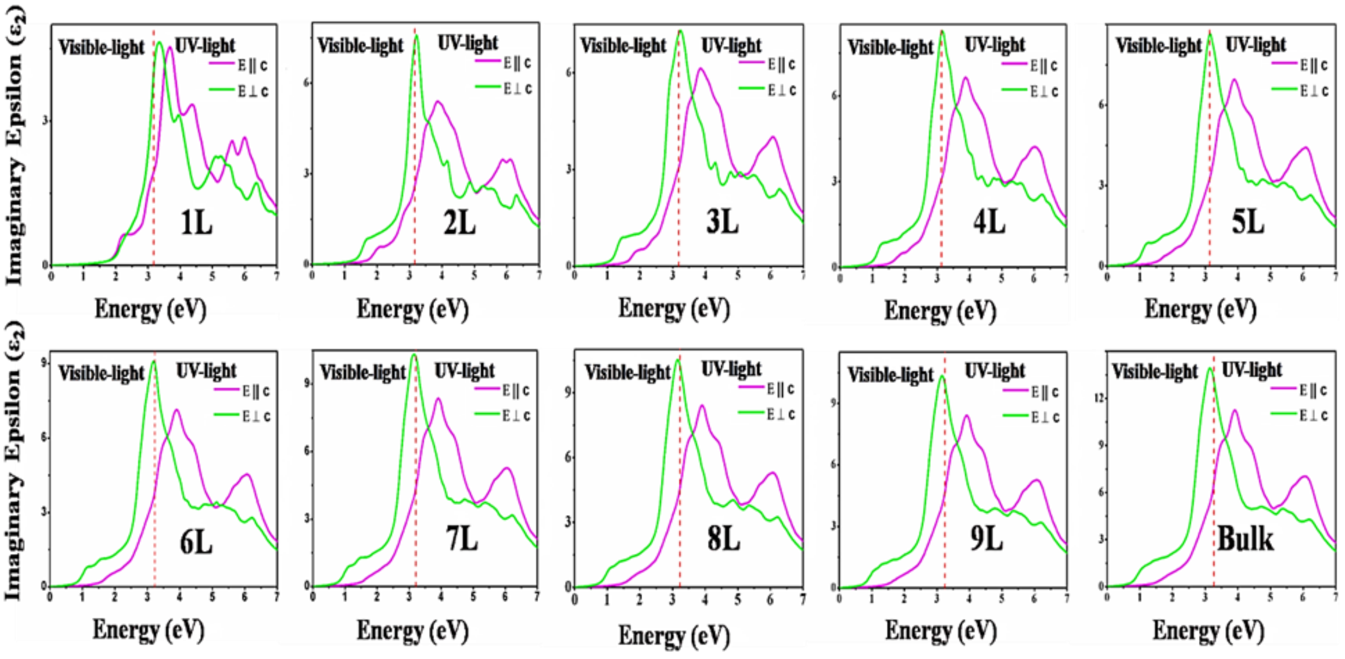


**Figure S6:** Calculated imaginary part of the dielectric function along x and z directions for β-InSe monolayer (1L), few-layer (2L to 9L) and bulk β-InSe.

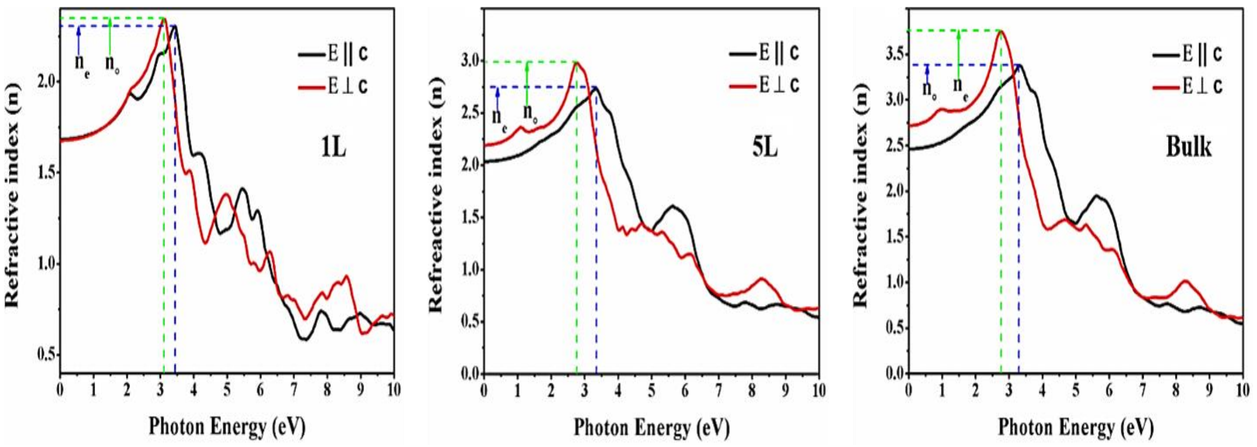


**Figure S7:** Calculated refractive index (n) along x and z directions for β-InSe monolayer (1L), few-layer (5L) and bulk β-InSe.
